# Fellow-Workers and the Roll of Honour

**Published:** 1915-10-09

**Authors:** 


### ROUND THE WORLD.

[The Editor Invites Early Information and Contributions Marked "Fellow-Workers."]

### Fellow-Workers and the Roll of Honour.

Recent fighting and the strenuous conditions of modern campaigning continue to give sad lists of doctors whose lives have been lost in the service of their country. This week we have no fewer than twelve names to mention, and that without any attempt to compile a complete roll of those who have fallen to date, so that other casualties in medical ranks will undoubtedly be reported shortly. To the Roll of Honour must now be added the following names as those of men whose work amongst us is finished gloriously: Captain A. E. Bullock, Lieutenant J. Clarke, Lieutenant C. M. Harris, Captain B. M. Hughes, Lieutenant J. McGowan, Lieutenant T. B. Paul, Lieutenant E. D. Parsons, Lieutenant J. W. Parker, Captain W. R. S. Roberts, Captain H. C. Storrie, Lieutenant P. J. Walsh, and Lieutenant F. J. Wisely.

Captain Arthur Ernest Bullock, R.A.M.C., was a young St. Mary's Hospital man who qualified in 1912, and subsequently held the posts of house surgeon, resident obstetric officer, and clinical assistant in the ophthalmic department at St. Mary's. The son of Dr. and Mrs. J. E. Bullock, of St. Leonards-on-Sea, he gained his captaincy in August 1914.

Lieutenant John Clarke, R.A.M.C. (T.F.), was practising in Wales when war broke out, and on volunteering for military work became posted to the 1st Welsh Field Ambulance. Ultimately he went to the Dardanelles, where, soon after arrival, he sustained the wounds which proved fatal. Mr. Clarke was by birth an Irishman, and received his education partly in Belfast and partly at Edinburgh University.

Lieutenant C. M. Harris, R.A.M.C., graduated at Sydney University only a few months ago, and was one of the batch of Australian doctors who answered the appeal for army surgeons in the spring. Being sent to Flanders, he was mortally wounded in one of the recent engagements.

Captain Burroughes Maurice Hughes died as a combatant officer, being attached to the Norfolk Regiment (4th Battalion). A St. Bartholomew's Hospital man, he qualified M.R.C.S., L.R.C.P. in 1895, and subsequently went through the Boer War, returning to private life with the rank of honorary lieutenant in the Army. For some little time Captain Hughes, who was forty-three years of age, has been living at Wymondham, in Norfolk.

Lieutenant Jeffery Wimperis Parker, R.A.M.C., was a New Zealander who had settled in practice at Penydaren, Merthyr Tydvil, after a general and medical education acquired at Dunedin, University College, Cardiff, and University College, London. Qualifying in 1906, he held resident posts at the East London Hospital for Children, Shadwell, and at the Addenbrooke Hospital, Cambridge. He joined the R.A.M.C. just a year ago.

Lieutenant Thomas Bond Paul, I.M.S., was serving with the Persian Gulf Expeditionary Force when attacked by fatal illness. A student of Middlesex Hospital, he qualified two years ago, and then, having held a house surgeoncy at that institution, he joined the Indian Medical Service just before mobilisation, and was stationed for awhile at Poona. He was a son of Dr. R. Paul, of Loughborough.

Lieutenant Edward Daniell Parsons, R.A.M.C., was doing good work in the Public Health Service when he decided to join the Army last November. Qualifying M.R.C.S., L.R.C.P. from St. Thomas's in 1903, he went for awhile to the Evelina Hospital for Sick Children as house physician. Then taking the D.P.H. he devoted his time to State medicine, and after acting as assistant M.O.H. and bacteriologist for Croydon he was appointed tuberculosis officer and deputy M.O.H. for Northampton. Leaving these posts for military duties, Mr. Parsons eventually was sent to Egypt, where he contracted the illness which was to send him home, and to end fatally in the 3rd London General Hospital at Wandsworth.

Captain Walter Rowland Southall Roberts, R.A.M.C. (T.F.), had made his home at Braintree on taking up the post of tuberculosis officer under the Essex County Council, to which he proceeded after public health experience as M.O.H. at Ongar and as medical officer at the West Ham Industrial School. The late officer went to the London Hospital after doing well at Birmingham University, where he was Queen's Scholar and Ingleby Scholar. Graduating M.B., Ch.B. of Birmingham in 1906, he subsequently took the diploma of the Conjoint Board, and worked as clinical assistant at the Hospital for Consumption, Brompton, and at the City of London Chest Hospital. On mobilisation he joined the 3rd East Anglian Field Ambulance, and this year went out to the Dardanelles, where he was killed.

Lieutenant Patrick Joseph Walsh, I.M.S., was an Irishman, who graduated M.B., B.Ch. of the Royal University of Ireland in 1912, after studying medicine at University College, Cork. Joining the Indian Medical Service in the following year, he was appointed medical officer to the 59th Scinde Rifles.

Lieutenant Francis Joseph Wisely, R.A.M.C., another medical officer mortally wounded in the Dardanelles campaign, was also an Irish graduate. After qualifying in 1911 he held resident appointments at Belfast, and then, entering the Lunacy Service, obtained the post of A.M.O. to the City and County Asylum at Powick, Worcestershire. Dr. Wisely, who joined the R.A.M.C. about this time last year, died in one of the Alexandria Hospitals.

The staff of the new City of London Red Cross Hospital is constituted as follows: Lieut.-Col. Thomas Openshaw, C.M.G., Mr. Hugh Lett, and Mr. R. Warren, surgeons; Dr. R. A. Young, Dr. Charles Buttar, and Dr. G. G. Howitt, physicians; Mr. Kenneth Scott, ophthalmologist; Mr. Norman Patterson, rhinologist and laryngologist; Dr. Edwin Ash, neurologist.

It will be noted that the London Hospital is strongly represented on the staff of this new Red Cross institution. Lieut.-Col. Openshaw and Mr. Lett are surgeons and joint lecturers on surgery at the "London," where Mr. Warren is assistant surgeon, and Mr. Patterson (assistant surgeon to the Golden Square Throat Hospital) is demonstrator and registrar in the throat department.

